# Diabetes in the Elderly

**DOI:** 10.1007/s13300-018-0380-x

**Published:** 2018-02-19

**Authors:** Sanjay Kalra, Suresh K. Sharma

**Affiliations:** 1 Department of Endocrinology, Bharti Hospital, Karnal, India; 2 College of Nursing, AIIMS, Rishikesh, India

**Keywords:** Diabetes education, Diabetes nursing, End of life, Geriatric, Geriatric syndromes, Hypoglycemia, Neurocognitive dysfunction

## Abstract

The elderly are an important and distinct yet heterogeneous group of persons living with diabetes. The elderly have a unique biomedical, psychological, and social constitution. Their needs are different from those of younger adults. This implies that special care must be taken while evaluating and planning their nursing and management. Diabetes management in the elderly should focus on prevention and limitation of geriatric syndromes (medical conditions encountered in elderly persons), hypoglycemia (low blood glucose), and neurocognitive dysfunction (impairment in the functioning of the nervous system and brain). This review takes a practical approach to the assessment, nursing care, and medical treatment of diabetes in the elderly. It highlights major challenges and suggests solutions to these commonly encountered clinical problems.

## Introduction

As the diabetes pandemic grows, and as persons with diabetes live longer, the prevalence of diabetes in the elderly is rising. The elderly constitute a significant proportion of all persons with diabetes. The elderly may seem to be a distinct and homogeneous class of individuals. In reality, however, the elderly are as heterogeneous as any other group of people. While many are as fit as younger adults, others experience challenges and limitations related to ageing. This review describes a practical approach to management of diabetes in elder persons who live with age-related medical and psychosocial health issues. The review also seeks to sensitize readers to deal with older persons in an appropriate manner. This article is based on previously conducted studies and does not involve any new studies of human or animal subjects performed by any of the authors. This practical approach paper will be reviewed on a biennial basis, or earlier if required, incorporating updates in the field of diabetes management in the elderly.

The definition of elderly or older is itself uncertain. While the International Diabetes Federation limits the use of this adjective to describe those above 70 years of age [1], the American Diabetes Association (ADA) includes all aged over 65 years as older [2]. The World Health Organization extends this label to persons who are 60 years or more of age [3]. The unique physiological constitution, biomedical needs, and psychosocial challenges of the elderly call for an individualized approach to their management. Such management should be tailored to the particular person’s requirements and should involve comprehensive interdisciplinary input from both nursing and medical care providers [4].

## Glucose Metabolism and Age

Diabetes is a disorder of glucose metabolism. It is not appreciated, however, that glucose metabolism varies with age in normal individuals as well. Glucose homeostasis, or balance, depends upon adequate insulin secretion from the pancreas and appropriate sensitivity of insulin receptors to the hormone. Both insulin secretion and insulin sensitivity are impaired with increasing age. Various factors frequently encountered in old age contribute to, or are associated with, insulin resistance. These include central obesity, induced by various environmental factors, secretion of AVP (arginine vasopressin) or its C-terminal fragment (copeptin). Vitamin D deficiency and hypomagnesemia have also been incriminated in the pathogenesis of diabetes in the elderly. Insulin secretion is impaired with advancing age as well [5].

Thus a 1–2 mg% increase in fasting blood glucose is noted with each decade. A 15 mg% rise in postprandial or post-challenge glucose levels is also seen after the third decade of life [6].

## Screening

Screening for diabetes is important in the elderly. The higher prevalence of diabetes in this age group suggests the need for a higher intensity of case-detection measures. The diagnostic criteria for diabetes are similar in the elderly and in young adults. However, screening strategies and semiology may vary.

Screening should be performed annually in the elderly, and opportunistic screening is recommended. This implies that plasma glucose should be checked whenever an elderly individual undergoes a routine or medically indicated blood test. A post-challenge or postprandial glucose test may be a better screening strategy than a fasting glucose estimation. Glycated hemoglobin, which assesses glucose control over the preceding 3 months, is a globally accepted parameter for diagnosis of diabetes. In the elderly, however, it has limited utility in diagnosis. This is because situations which affect red blood cell life-span (anemia, acute illness) occur frequently in the elderly [7].

## Symptomatology

In the healthy person, glucose is reabsorbed by the kidney to ensure normal levels of glucose in the blood. In a person with diabetes, glucose is excreted through the kidney if it crosses a particular level, termed the renal threshold. The renal threshold for glucose increases with age, and thirst mechanisms are impaired. Thus the symptoms of polyuria (excessive urination) and polydipsia (excessive thirst) may not present early in elderly persons with diabetes. Patients may present with easy fatigability, refractory or recurrent infections, weight loss, or chronic vascular complications.

Most of the common geriatric syndromes may be due to diabetes. Geriatric syndromes are commonly encountered clinical situations that have a substantial impact on functionality and quality of life in the elderly. These include pressure ulcers, incontinence, falls, functional decline, and delirium. Other geriatric syndromes that are accepted as such include dementia, hearing impairment, visual impairment, sarcopenia, malnutrition, frailty, immobility, and gait disturbance (Table 1). Four risk factors (older age, baseline cognitive impairment, baseline functional impairment, and impaired mobility) contribute to the majority of geriatric syndromes [8]. Diabetes can contribute to all these syndromes (Table 1), either directly through its symptoms and complications or by delaying resolution of other comorbid conditions.

**Table 1:** Geriatric syndromes in the elderly with diabetes

Hyperglycemia/metabolic decompensation
Hypoglycemia
Cognitive impairment
Motor functional impairment
Falls
Visual impairment
Auditory impairment
Psychosocial impairment
Polypharmacy
Dependency

## Comprehensive Geriatric Assessment

A comprehensive geriatric assessment is mandatory while treating elderly persons with diabetes [9]. A geriatric assessment implies a careful clinical analysis of the individual to determine how fit he or she is to manage self-care activities. This includes an analysis of basic, independent, and advanced activities of daily living which can be carried out by the individual. On the basis of such triage, the elder person can be classified according to functional status, comorbid conditions, and expected life-span. This assessment helps plan supportive and therapeutic strategies. Table 2 provides an overview of how to perform a triage for elder persons with diabetes. The risk of hypoglycemia should be evaluated in every person: this is one of the most important factors in ensuring an appropriate therapeutic choice.

**Table 2:** Therapeutic targets for the elderly with diabetes

Triage category	1	2	3
Overall health	Good	Intermediate	Poor
Health challenges	Few	Complex	Very complex
Risk of hypoglycemia	Low	Intermediate	High
Comorbid illness	Few	Multiple (≥ 3)	End-stage chronic disease
Cognitive status	Preserved	Mild–moderate impairment	Moderate–severe impairment
Functional status	Preserved	Impairment in ≥ 2 ADL	Dependency in ≥ 2 ADL
Life expectancy	Long	Intermediate	Limited
Treatment burden	Low	High	Variable
Self-motivation	Good	Intermediate	Poor
Family/social support	Good	Intermediate	Poor
HbA1c goal	< 7.5%	< 8.0%	< 8.5%
Fasting plasma glucose goal	90–130 mg%	90–150 mg%	100–180 mg%
Bedtime glucose goal	90–150 mg%	100–180 mg%	110–200 mg%
Blood pressure	< 140/90 mmHg	< 140/90 mmHg	< 150/90 mmHg

Three specific issues need attention in the geriatric diabetes context. These are neurocognitive dysfunction, hypoglycemia, and psychosocial issues.

Neurocognitive dysfunction, or impairment in the functioning of the nervous system and brain, is common in the elderly. The severity of dysfunction may vary from subtle impairment in memory to full-blown Alzheimer’s disease or memory loss. An annual screening must be carried out to assess neurocognitive function. Elderly persons with neurocognitive challenges should be prescribed simple regimens of antidiabetic therapy, with lesser frequency, flexible timing, and easy method of administration. The heterogeneity of the elderly, as discussed earlier in this article, manifests in the versatility of glucose-lowering prescriptions as well.

Hypoglycemia, or low blood glucose, is a common occurrence in diabetes care [10]. The elderly are especially prone to this complication [11]. Hypoglycemia awareness (the ability to recognize symptoms of low blood glucose) is limited, and glucagon and epinephrine response to hypoglycemia (the endocrine defense mechanism to correct low blood glucose) are blunted in the elderly. Hypoglycemia awareness training (training individuals with diabetes to recognize, limit, manage, and prevent low blood glucose) is, therefore, an integral part of diabetes care for the elderly. Regimens and drugs with a low risk of hypoglycemia must be preferred in this age group.

Apart from their biomedical complications, the elderly also face unique psychosocial challenges. These include a higher risk of depression and an unfriendly social environment. The psychosocial support available to the person with diabetes must be assessed as part of routine clinical work. Screening for quality of life, diabetes distress, and depression should also be performed using simple validated tools.

## Aim of Management

The main aim of providing diabetes care to the elderly is to achieve comfort, optimal quality of life, resolution of symptoms, and avoid acute complications. Thus, numerical targets are not as important as symptomatic well-being. HbA1c targets can be relaxed to < 7.5%, < 8.0%, and 8.5% in active, fairly healthy, and terminal elderly persons with diabetes, respectively [2]. Similarly, preprandial (before meal) and postprandial (after meal) glycemic targets suggested by the ADA are listed in Table 2.

Management of associated metabolic dysfunction is part of comprehensive care. This includes a focus on blood pressure, weight, and lipid levels. Blood pressure control is important, but systolic blood pressure targets can be relaxed to 150/90 mmHg in persons with limited life expectancy and multiple comorbidities [2]. Here too, the emphasis is on maintenance of a symptom-free life and avoidance of iatrogenic postural hypotension [12].

## Tools for Management

The non-pharmacological and pharmacological tools available for management of diabetes are the same for elderly and young adults [13]. The subtle differences in medical approach to diabetes care are listed in Table 3. In general, elderly persons respond to lower doses and do not require aggressive glucose-lowering therapy.

**Table 3:** Preferred medical therapy in elderly with diabetes

General approach
While diagnostic criteria are similar for adults of all ages, glycemic targets may be relaxed for the elderly
Follow the same hierarchy of choosing a glucose-lowering therapy as recommended for younger adults
Non-insulin drugs
Metformin is the drug of choice along with lifestyle modification, provided it is not contraindicated and is well tolerated
DPP4 inhibitors are preferred owing to the lower risk of hypoglycemia
Modern sulfonylurea may be used in low doses. Glibenclamide should be avoided
Pioglitazone should be avoided because of the risk of fractures and heart failure
SGLT2 inhibitors may be used in otherwise healthy persons with adequate nutrition and hydration while keeping vigilance for complications
GLP1RA may be used, provided the injectable therapy is accepted and tolerated
Insulin therapy
The indications for insulin are similar in adults of all age groups
If basal insulin is required, prefer preparations with a lower risk of hypoglycemia and nocturnal hypoglycemia, such as degludec and U300 glargine
If prandial coverage is necessary, prefer premixed insulin analogues or coformulations with a lower risk of hypoglycemia and nocturnal hypoglycemia, e.g., biphasic aspart, biphasic lispro, insulin degludec aspart
Low mixtures should be preferred over high mixtures
Once-daily regimens should be preferred over twice-daily regimens
Pen devices should be preferred over syringes and vials for insulin delivery

## Nursing Issues

Diabetes in the elderly requires multidisciplinary management, which includes other providers, when available, such as dieticians, physical therapists for balance and gait to avoid falls, social work, as well as the involvement of the patient and family. The remainder of this article highlights the aspects which need attention. While the focus is on nursing care, many of these issues are better addressed if specialist healthcare professionals such as dieticians and physiotherapists are available. Some of the main nursing challenges in the elderly are listed in Table 4. The remainder of this article focuses on a practical approach to nursing the elderly with diabetes. It includes discussion related to indoor nursing, as well as home or community nursing.

**Table 4:** Nursing issues in the elderly

Lifestyle management
Hydration
Nutrition
Physical activity and motility
Stress management
Therapeutic support
Therapy administration
Glucose monitoring
Dose titration
Hypoglycemia prevention and care
Complication prevention/care
Muscle mass maintenance
Foot care
Prevention of falls
Pressure ulcer care

## Nutrition and Hydration

Nutrition and hydration may be impaired in the elderly with diabetes. Alteration in taste, reduced appetite due to drugs, difficulty in chewing, gastric irritation, and gastrointestinal dysmotility or malabsorption may compromise nutritional status. Dysmotility may present as diarrhea, constipation, or frequent change in bowel habits. Dysfunctional thirst mechanisms may act as an obstacle to adequate fluid intake, thus leading to dehydration.

Nursing care should ensure that the basic needs of nutrition and hydration are not neglected. The “eight As” remind the nursing practitioner of the important considerations in diet planning. The diet prescription should not only be accurate but also appropriate. The diet should be available/accessible, acceptable, attractive, achievable, and affordable. Advised meals should be absorbable/digestible as well [14]. A rule of thumb to help ensure optimal fluid intake is 10 ml/kg for the first 10 kg body weight, 50 ml/kg for the next 10 kg, and 15 ml/kg for further weight.

## Physical Activity and Mobility

Physical activity is an essential part of diabetes management. In the elderly, maintenance of mobility along with regular physical activity is an integral part of nursing care. Such exercises help prevent and reduce the risk of sarcopenia, or muscle loss. This, in turn, helps prevent falls and fractures as well. Flexibility exercises and yoga, along with mobility exercises, should be integrated into diabetes care and encouraged. Those at risk of osteoporosis should avoid high impact exercises. Low impact exercises, in which at least one foot is on the ground at all times, may be practiced.

## Stress Management and Cognitive Function

Stress management is as important in the elderly as it is in other age groups. Nursing caregivers should be sensitive to the psychosocial needs and challenges of the elderly. Available opportunities must be utilized to improve coping skills and stress management. Non-pharmacological strategies to preserve cognitive function and delay dementia or memory loss may be tried.

## Prevention of Ulcers and Falls

Nursing care in the elderly with diabetes also includes interventions for foot health, prevention of falls and fractures, and prevention of pressure ulcers. Nursing should focus not only on micromanagement in a given individual but also macromanagement of the environment. Changes in footwear, lifestyle, furniture, bedding, and architecture contribute significantly to mitigation of risk factors for ulcers and falls. The diabetes nurse should work as an advocate for the provision of a diabetes-friendly environment and society.

## Therapy Administration and Monitoring

Therapeutic support is an integral part of diabetes nursing. Insulin is usually self-administered, but elderly persons with visual, tactile, or motor impairment may need nursing support in insulin injection. Similar support may be necessary for glucose monitoring and dose titration. Nursing care providers must focus on avoidance of hypoglycemia. The ASAP (anticipate, suspect, act, prevent) strategy is a useful guide for diabetes nursing [11].

Modern insulin delivery devices are available which can be used easily by visually and functionally challenged older adults [15]. Examples include pens with large-sized numbers, audible clicks on dose dialing, and low-pressure requirement for administration. These can be utilized with patient-friendly regimens and insulin preparations, which achieve good glycemic control with minimal risk of hypoglycemia and unwanted weight gain. “Weekend” therapy in the form of once-weekly injectable glucagon-like peptide 1 receptor agonist (GLP1RA) (dulaglutide, exenatide) is also available [16]. This obviates the need for frequent injections. Subcutaneous implants of exenatide, which can provide effective control for up to 12 months, are in advanced stages of development.

## Polypharmacy

Polypharmacy is a reality of modern evidence-based diabetes care. However, this brings with itself a heightened risk of adverse effects, drug–drug interactions, and iatrogenic (caused by medical care) complications. These can be minimized by following the law of therapeutic parsimony [17]. This law states that minimum drug preparations and doses should be used, as long as they are able to achieve optimal outcomes. Current guidelines for management of the elderly with diabetes promote relaxed targets and avoidance of aggressive control.

## End of Life Care

At the terminal end of life, the goals of treatment change from number-based targets to symptomatic well-being and preservation of dignity/quality of life. Through a process of shared decision-making, including the person with diabetes, family, nursing and medical team, one may reduce the intensity of therapy and monitoring. In stable patients, one may continue the previous regimes, with a focus on preventing hypoglycemia and symptomatic hyperglycemia. Monitoring of urine glucose is more relevant than HbA1c at this stage of life. However, it must be noted that renal threshold for glycosuria rises with age.

In persons with organ failure, the main goal is to prevent hyperglycemia and dehydration. Drug doses may have to be down-titrated and complex insulin regimens discontinued. Modern insulin analogues, with low risk of hypoglycemia and nocturnal hypoglycemia, may be used in such situations. In dying persons with type 2 diabetes with minimal oral intake, insulin may be discontinued. In dying persons with type 1 diabetes, basal insulin may be administered in small doses to prevent acute metabolic decompensation [2, 4].

## Summary

The elderly represent a distinct yet heterogeneous group of persons with diabetes. Their unique physiology, pathophysiology, clinical features, needs, and challenges suggest that they need individualized diabetes care. On the basis of their functional ability and medico-surgical comorbidities, the elderly aim for relaxed targets, with a strategy to remain cognizant of geriatric syndromes, and avoid hypoglycemia. Tools or interventions used must be safe, well tolerated, and easy to administer while requiring minimal monitoring.

## References

[1] Dunning T, Sinclair A, Colagiuri S (2014). New IDF guideline for managing type 2 diabetes in older people. Diabetes Res Clin Pract.

[2] American Diabetes Association (2018). 11. Older adults: standards of medical care in diabetes—2018. Diabetes Care.

[3] World Health Organization (2015). World report on ageing and health.

[4] Munshi MN, Florez H, Huang ES (2016). Management of diabetes in long-term care and skilled nursing facilities: a position statement of the American Diabetes Association. Diabetes Care.

[5] Chentli F, Azzoug S, Mahgoun S (2015). Diabetes mellitus in elderly. Indian J Endocrinol Metab.

[6] Baruah MP, Kalra S, Unnikrishnan AG (2011). Management of hyperglycemia in geriatric patients with diabetes mellitus: South Asian consensus guidelines. Indian J Endocrinol Metab.

[7] Bajwa SJ, Sehgal V, Kalra S, Baruah MP (2014). Management of diabetes mellitus type-2 in the geriatric population: current perspectives. J Pharm Bioallied Sci.

[8] Kalra S (2013). Geriatric diabetes. J Pak Med Assoc.

[9] American Geriatrics Society Expert Panel on the Care of Older Adults with Diabetes Mellitus (2013). Guidelines abstracted from the American Geriatrics Society guidelines for improving the care of older adults with diabetes mellitus: 2013 update. J Am Geriatr Soc.

[10] Seaquist ER, Anderson J, Childs B (2013). Hypoglycemia and diabetes: a report of a workgroup of the American Diabetes Association and the Endocrine Society. J Clin Endocrinol Metab.

[11] Kalra S, Gupta Y (2017). Prevention of hypoglycaemia, the ASAP (anticipate, suspect, act, prevent) strategy. J Pak Med Assoc.

[12] Rich MW, Chyun DA, Skolnick AH (2016). Knowledge gaps in cardiovascular care of the older adult population: a scientific statement from the American Heart Association, American College of Cardiology, and American Geriatrics Society. J Am Coll Cardiol.

[13] Mitrakou A, Katsiki N, M Lalic N (2017). Type 2 diabetes mellitus and the elderly: an update on drugs used to treat glycaemia. Curr Vasc Pharmacol.

[14] Kalra S, Kalra B, Saluja S. Dietary management in geriatric patients of diabetes mellitus: special considerations. Internet J Geriatr Gerontol. 2009;5(1).

[15] Tandon N, Kalra S, Balhara YP (2017). Forum for injection technique and therapy expert recommendations, India: the Indian recommendations for best practice in insulin injection technique, 2017. Indian J Endocrinol Metab.

[16] Kalra S, Gupta Y (2016). Weekend therapy in diabetes. J Pak Med Assoc.

[17] Kalra S, Gupta Y, Sahay R (2016). The law of therapeutic parsimony. Indian J Endocrinol Metab.

